# Cognitive factors influenced physical distancing adherence during the COVID-19 pandemic in a population-specific way

**DOI:** 10.1371/journal.pone.0267261

**Published:** 2022-05-03

**Authors:** Gillian A. M. Tarr, Keeley J. Morris, Alyson B. Harding, Samuel Jacobs, M. Kumi Smith, Timothy R. Church, Jesse D. Berman, Austin Rau, Sato Ashida, Marizen R. Ramirez

**Affiliations:** 1 Division of Environmental Health Sciences, School of Public Health, University of Minnesota, Minneapolis, MN, United States of America; 2 Division of Epidemiology & Community Health, School of Public Health, University of Minnesota, Minneapolis, MN, United States of America; 3 Department of Community and Behavioral Health, College of Public Health, University of Iowa, Iowa City, IA, United States of America; Emory University, School of Public Health, UNITED STATES

## Abstract

Even early in the COVID-19 pandemic, adherence to physical distancing measures was variable, exposing some communities to elevated risk. While cognitive factors from the Health Belief Model (HBM) and resilience correlate with compliance with physical distancing, external conditions may preclude full compliance with physical distancing guidelines. Our objective was to identify HBM and resilience constructs that could be used to improve adherence to physical distancing even when full compliance is not possible. We examined adherence as expressed through 7-day non-work, non-household contact rates in two cohorts: 1) adults in households with children from Minnesota and Iowa; and 2) adults ≥50 years-old from Minnesota, one-third of whom had Parkinson’s disease. We identified multiple cognitive factors associated with physical distancing adherence, specifically perceived severity, benefits, self-efficacy, and barriers. However, the magnitude, and occasionally the direction, of these associations was population-dependent. In Cohort 1, perceived self-efficacy for remaining 6-feet from others was associated with a 29% lower contact rate (RR 0.71; 95% CI 0.65, 0.77). This finding was consistent across all race/ethnicity and income groups we examined. The barriers to adherence of having a child in childcare and having financial concerns had the largest effects among individuals from marginalized racial and ethnic groups and high-income households. In Cohort 2, self-efficacy to quarantine/isolate was associated with a 23% decrease in contacts (RR 0.77; 95% CI 0.66, 0.89), but upon stratification by education level, the association was only present for those with at least a Bachelor’s degree. Education also modified the effect of the barrier to adherence leaving home for work, increasing contacts among those with a Bachelor’s degree and reducing contacts among those without. Our findings suggest that public health messaging tailored to the identified cognitive factors has the potential to improve physical distancing adherence, but population-specific needs must be considered to maximize effectiveness.

## Introduction

Physical distancing has been and will continue to be an important strategy in managing COVID-19 until the “next normal” emerges; i.e., when mortality rates and immunity levels are comparable to other respiratory viruses [1]. It remains particularly important for protecting vulnerable populations such as underrepresented racial/ethnic communities [2–4]. While the effectiveness of physical distancing for disease control is well supported by observational [5–7] and modelling studies [8–13], effectiveness relies on community-level adherence [14, 15].

Non-adherence to physical distancing guidelines, such as self-isolation, quarantine, and “lockdowns” is highly variable and can exceed 90% depending on the stringency of the definition [16–19]. Adherence can be challenging because of the many difficulties it imposes on individuals, including job loss, social isolation, and negative mental health effects [20–23]. Studies suggest a variety of factors have contributed to differential adherence to physical distancing guidelines during the COVID-19 pandemic. These include sociodemographic factors, such as age [24–26], income [27], gender [24, 26, 28], and education [19], as well as health and psychosocial factors, such as mental health status [26, 28], physical health status [19, 26], information sources [28], personal and societal norms [19, 24, 29], and perceptions of government handling of the response [19, 26].

Behavioral theories suggest that motivation to adhere to protective behaviors like physical distancing depend on numerous individual and contextual factors. Among these is the Health Belief Model (HBM) [30, 31], which outlines a set of constructs that could be targeted in public health communication to improve health behaviors. Others have demonstrated how the HBM can be applied to COVID-19 public health messaging [32, 33], including how it can be tailored for COVID-19 risk communication strategies based on sociodemographic identities [34]. While not a part of the HBM, the concept of resilience, both at the personal [35] and community [36] levels, is important to how individuals respond to stressors, such as the pandemic. Both resilience and cognitive factors within the HBM have proven to be good predictors of pandemic-related health behaviors or behavioral intent, including compliance with physical distancing-related best practices (e.g. working from home) [37–44]. However, physical distancing is not an all-or-nothing behavior as assessed in these studies, and external factors may prevent full compliance with physical distancing guidelines. Even if full compliance is not possible, some degree of adherence, and therefore risk reduction, likely is. It remains unclear which of the HBM and resilience constructs are best suited to leverage for improving adherence to physical distancing, particularly across heterogeneous population subgroups.

Here, we show that resilience and several cognitive factors from the HBM are associated with physical distancing adherence as measured through non-work, non-household contact rates; however, these associations are often population-dependent. Surveying two cohorts, we found that perceived severity, benefits, and self-efficacy were associated with lower numbers of contacts, and community resilience and perceived barriers were associated with greater numbers of contacts. Race/ethnicity and socioeconomic status magnified, and in some cases reversed, some associations. Based on these findings, we conclude that public health messaging based on cognitive factors has the potential to improve physical distancing adherence, but communications must be population-specific.

## Materials & methods

### Study population

Participants were enrolled in the COVID-19 Preparedness & Response Study between April 16, 2020 and June 9, 2020, a period of stay-at-home orders, non-essential business and school closures, and other similarly restrictive public health guidance. Participants were recruited into one of two study cohorts that could experience particular challenges to physical distancing adherence. Cohort 1 (C1), the “family cohort,” was recruited via social media (Facebook, Twitter); participants came from Minnesota and Iowa and were limited to adults living in households with children <18 years old. Cohort 2 (C2), the “older adult cohort,” was recruited from an established registry for the study of Parkinson’s disease; participants came from Minnesota and were limited to adults ≥50 years old.

This study was approved by the University of Minnesota Institutional Review Board (IRB) under STUDY00009362. Consent was obtained via an online form or verbally over the phone with approval of the IRB.

### Survey

Participants completed an online REDCap [45] survey, with the exception of 7.4% (25/339) of participants in the older adult cohort who requested to complete the survey by telephone; trained interviewers entered their data into a REDCap form.

#### Outings and contacts

To measure adherence to physical distancing, participants were asked to report the frequency of visits to seven types of locations during non-work outings in the seven days before the survey (S1 Appendix). For each type of outing made in the past seven days, participants reported the number of non-work, non-household contacts they had on their most recent outing. They also provided the number of visitors to their homes during the prior seven days.

Our primary outcome was the overall number of non-work, non-household contacts (including visitors) over the previous 7 days. We multiplied the number of contacts during the most recent outing by the number of outings in the prior seven days for each outing type. Total contacts in the prior 7 days was then obtained by summing contacts for all outing types and visitors to the home.

#### Health Belief Model (HBM)

**Fig 1:**
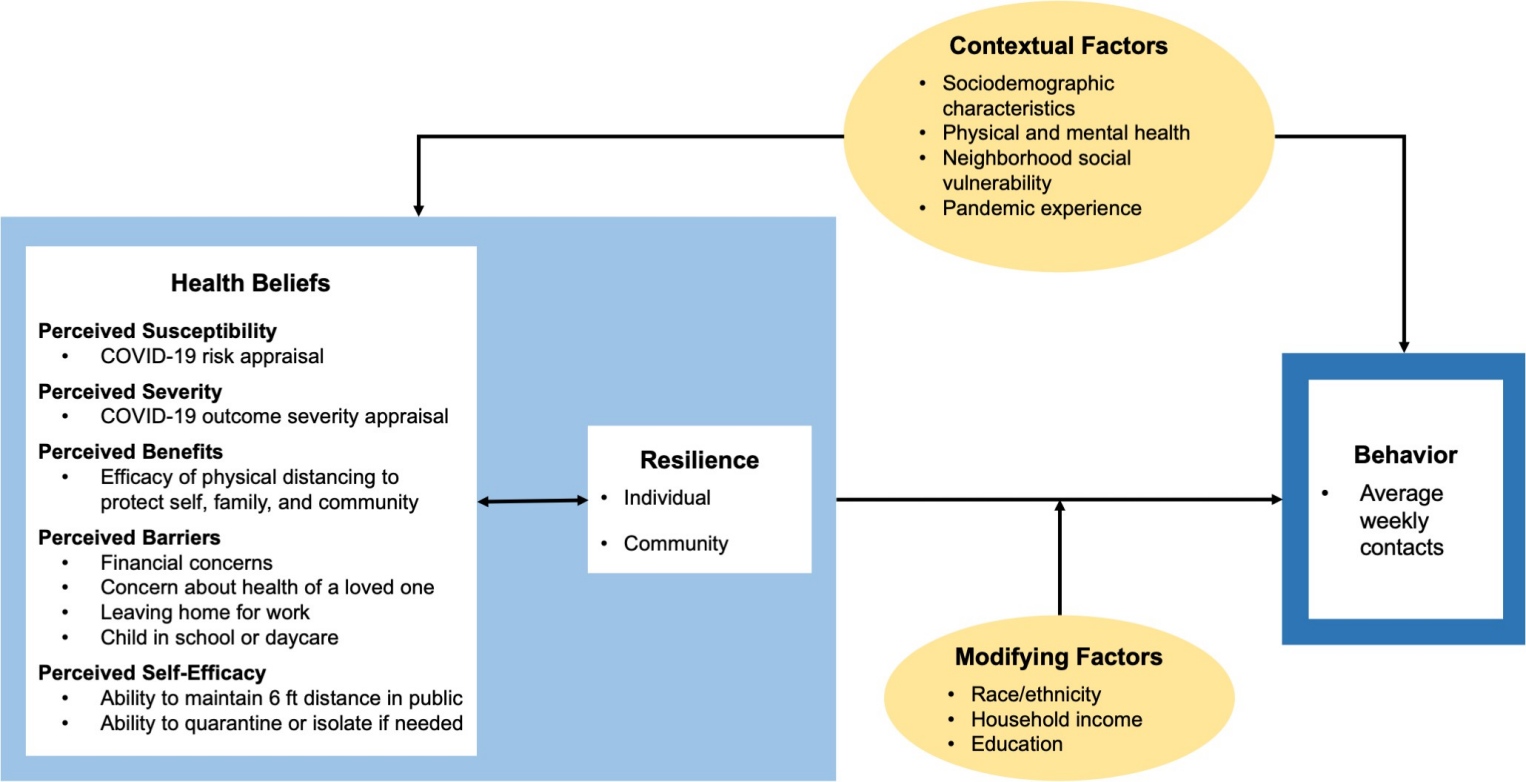
Conceptual model relating cognitive factors from the Health Belief Model and resilience to pandemic non-work, non-household contact rates. The light blue box shows the cognitive factors from the Health Belief Model, ‘Health Beliefs’, and resilience constructs that we evaluated and the variables we used to assess them. Contextual factors were assessed as potentially confounding the association between cognitive factors/resilience and contacts, and modifying factors were considered to modify the association.

We adapted the Health Belief Model (HBM), including the self-efficacy construct [30, 31], to identify cognitive factors associated with adherence to physical distancing, as expressed through elevated contact rates (Fig 1).

We assessed participants’ perceived risk and severity specific to COVID-19 on 5-point Likert scales from Very High to Very Low, which were reversed for analysis (Table A in S1 Appendix). Perceived benefits were measured as the response efficacy of physical distancing at the individual, household, and community levels measured using a 5-point scale from Strongly Disagree to Strongly Agree (Table A in S1 Appendix). Exploratory factor analysis (EFA) identified these three response efficacy questions as grouped; their values were averaged to make a composite perceived benefits score.

Perceived self-efficacy was based on direct self-assessments of self-efficacy for physical distancing behaviors: maintaining 6 feet of distance from non-household members while in public and quarantining or isolating as needed. These were measured using a 4-point scale from ‘Not confident at all’ to ‘Very confident’ (Table A in S1 Appendix).

Perceived barriers were assessed using a series of statements such as “I don’t have enough money to pay for my basic needs right now,” assessed on a 5-point scale from Strongly Disagree to Strongly Agree (Table A in S1 Appendix). Situational factors, including needing to leave home for work and having a child in daycare or school, were used as proxies for additional perceived barriers.

A range of contextual factors were assessed (Fig 1), including sociodemographics, mental and physical health characteristics, and neighborhood characteristics (S1 Appendix).

#### Resilience

Individual resilience was measured by the two-item Connor-Davidson Resilience Scale (CD-RISC2), an instrument that has been reported to be highly correlated (r = 0.78, P<0.001) with the full 25-item CD-RISC [46]. Community resilience was measured by the ten-item Conjoint Community Resiliency Assessment Measure (CCRAM), which has a reported internal consistency of α = 0.85 [47] (S1 Appendix).

### Analysis

Perceived barriers and potential confounders were examined in descriptive analyses to determine inclusion in subsequent models. For categorical variables, we compared the median and 25^th^ and 75^th^ quantiles of contacts across strata. For continuous variables, we inspected graphs of total contacts vs. the variable with a cubic spline smoother with the maximum number of knots.

To identify HBM and resilience constructs associated with non-work, non-household contact rates in each cohort, we used negative binomial models given the preponderance of participants with no contacts. Because barriers during the pandemic could be highly situational, only those barriers appearing to have some relationship with contacts in the descriptive analyses were included in the negative binomial models. Models were adjusted *a priori* for age, gender, race, Parkinson’s disease (C2 only), and a measure of SES (C1: income; C2: education). Other potential confounders identified based on the descriptive analyses were included if their inclusion did not cause >10% of participants to be excluded from the analysis due to missing data. Model coefficients were exponentiated to obtain rate ratios (RRs) for the rate of contacts in the previous seven days associated with a one-unit increase in the given covariate.

#### Secondary analysis

To determine whether sociodemographic factors modify the effect of cognitive factors or resilience on contact rates, we stratified our models by race/ethnicity in C1 (insufficient diversity to do so in C2) and measures of socioeconomic status in both cohorts (Fig 1). If the 95% CI of one stratum did not overlap the effect estimate of another stratum, and at least one was a meaningful and significant effect, we considered the variation of interest (S1 Appendix).

## Results

Our primary goal was to identify opportunities to improve adherence to physical distancing during periods of high community transmission. We approached this using resilience and the Health Belief Model (HBM) to examine cognitive factors affecting non-work, non-household contacts in two cohorts. We subsequently stratified our full models by key sociodemographic modifiers to understand how key perceptions and resilience impact physical distancing behavior differently among some populations.

### Cohort 1 (C1): Families in Minnesota and Iowa

We consented 1,105 participants in C1, 997 (90%) of whom completed the survey. Approximately two-thirds of C1 participants lived in Minnesota, with the other third in Iowa (Table 1). The average of this cohort was 40.6 (SD 6.5) years, and 89% identified as women. Approximately 1.1–2.7% of participants identified as Black, Hispanic/Latinx, American Indian or Alaska Native, and Asian, relative to 2.0–6.6% and 1.3–5.0% of the Minnesota and Iowa populations, respectively. A household income >$80,000 was reported by 71% of C1 participants, relative to median family incomes of $93,584 in Minnesota and $78,152 in Iowa.

**Table 1:** Participant characteristics by study cohort, with source state population profiles provided for comparison.

	Family Cohort (C1) N = 997	Older Adult Cohort (C2) N = 339	Minnesota Population Profile ^a^	Iowa Population Profile ^a^
**Age (years), n (%)**				
** 18–35**	173 (17.4)	0	22.3%	22.5%
** 35–44**	573 (57.5)	0	13.0%	12.4%
** 45–54**	226 (22.7)	7 (2.1)	11.9%	11.4%
** 55–64**	23 (2.3)	101 (30.0)	13.4%	13.3%
** 65–74**	2 (0.2)	163 (48.1)	9.5%	9.9%
** ≥75**	0	68 (20.1)	6.9%	7.7%
**Gender identity, n (%)**				
** Man**	107 (10.7)	163 (48.1)	49.7%	49.8%
** Woman**	882 (88.5)	173 (51.0)	50.3%	50.2%
** Transgender or Non-binary**	5 (0.1)	1 (0.3)	NR	NR
** Not provided**	3 (0.3)	2 (0.6)		
**Racial/ethnic identity ^b^, n (%)**				
** American Indian or Alaska Native**	21 (2.1)	5 (1.5)	2.0%	1.3%
** Asian**	27 (2.7)	0	5.4%	2.7%
** Black**	11 (1.1)	0	6.6%	4.1%
** Hispanic/Latinx**	16 (1.6)	1 (0.3)	5.5%	5.0%
** Middle Eastern**	3 (0.3)	0	NR	NR
** Pacific Islander**	1 (0.1)	0	0.1%	0.3%
** White**	955 (95.8)	333 (98.2)	82.8%	87.9%
** Other**	8 (0.8)	4 (1.2)	1.2%	0.9%
** Not provided**	4 (0.4)	3 (0.9)		
**Number in household, mean (SD)**	4.21 (1.13)	2.33 (2.15)	All: 2.48 Families: 3.09	All: 2.38 Families: 2.96
**Education, n (%)**				
** Less than high school**	2 (0.2)	0	6.4%	7.4%
** High school**	39 (3.9)	11 (3.2)	24.4%	31.0%
** Post high school, including trade school**	134 (13.4)	80 (23.6)	31.9%	32.3%
** 4-year college degree**	391 (39.2)	131 (38.6)	24.5%	19.8%
** Masters/doctorate**	428 (42.9)	115 (33.9)	12.7%	9.5%
** Not provided**	3 (0.3)	2 (0.6)		
**Income, n (%)**			Median household income - All: $74,593 Families: $93,584	Median household income - All: $61,691 Families: $78,152
** Under $60,000**	133 (13.3)	71 (20.9)		
** $60,000-$79,999**	135 (13.5)	43 (12.7)		
** $80,000 or above**	708 (71.0)	197 (58.1)		
** Don’t know**	13 (1.3)	11 (3.2)		
** Not provided**	8 (0.8)	17 (5.0)		
**Parkinson’s disease, n (%)**	-	113 (33.3)		
**State, n (%)**				
** Iowa**	342 (34.3)	0		
** Minnesota**	655 (65.7)	339 (100)		

^a^ State data was abstracted from the 2019 American Community Survey (all but race and ethnicity) and 2020 Decennial Census (race and ethnicity).

^b^ Does not add to 100%, because respondents were able to select more than one racial or ethnic identity.

Abbreviations: NR, not reported; SD, standard deviation.

### Cohort 2 (C2): Older adults in Minnesota

We consented 358 participants in C2, 339 (95%) of whom completed the enrollment survey. The average age was 68.2 (SD 7.3) years, 51% were women, and one-third had Parkinson’s disease (Table 1). Five participants (1.5%) identified as American Indians or Alaskan Natives, compared to 2.0% statewide. Individuals from other racially and ethnically marginalized populations were mostly absent from the cohort. Compared to 37% of Minnesotans, 73% of C2 participants had attained a Bachelor’s degree or higher.

The average number of non-work, non-household contacts in the prior seven days was 4.9 (SD 5.9; range 0–45) and 5.8 (SD 6.1; range 0–37) for participants in C1 and C2, respectively.

### Multiple Health Belief Model (HBM) cognitive factors were associated with contact rates

To identify opportunities for improving adherence to physical distancing, we analyzed cognitive factors identified in the HBM using adjusted negative binomial models (Table B in S1 Appendix). Perceived severity, benefits (Cronbach’s α = 0.91), and all forms of perceived self-efficacy and barriers examined were associated with contact rates in the family cohort, but only self-efficacy to quarantine or isolate was associated with contact rates in the older adult cohort (Table 2). In C1, self-efficacy to stay 6-feet away from others while in public exhibited the strongest association with lower contact rates. Each 1-point increase in self-efficacy on this item was associated with a 29% decrease in the number of contacts over the previous 7 days (RR 0.71; 95% CI 0.65, 0.77). Self-efficacy to quarantine or isolate as needed was also associated with a 10% reduction in contacts in C1 (RR 0.90; 95% CI 0.82, 0.98) and 23% in C2 (RR 0.77; 95% CI 0.66, 0.89). These results indicate that self-efficacy had the largest positive association with adherence of the HBM cognitive factors in both cohorts.

**Table 2:** Results of negative binomial models testing the effect of Health Belief Model (HBM) and resilience constructs on non-work, non-household contact rates during April and May 2020.

	Cohort 1: Adults with Children ^a^		Cohort 2: Older Adults ^b^	
	*RR*	*95% CI*	*RR*	*95% CI*
**Health Belief Model Perceptions**				
Risk	1.07	(0.98, 1.16)	0.97	(0.84, 1.11)
Severity	0.89	(0.82, 0.97)	0.94	(0.82, 1.07)
Benefits	0.86	(0.78, 0.93)	0.86	(0.74, 1.00)
Self-efficacy: Distancing	0.71	(0.65, 0.77)	0.95	(0.82, 1.10)
Self-efficacy: Quarantine	0.90	(0.82, 0.98)	0.77	(0.66, 0.89)
Barrier: Leaves home for work	1.20	(1.01, 1.41)	1.28	(0.92, 1.79)
Barrier: Child in daycare/school	1.37	(1.09, 1.72)		
Barrier: Concern about finances	1.15	(1.05, 1.25)		
**Resilience**				
Individual resilience	1.02	(0.96, 1.09)	1.03	(0.93, 1.15)
Community resilience	1.26	(1.11, 1.43)	1.09	(0.86, 1.39)
N	925		309	

^a^ Cohort 1 model adjusted for age (linear and quadratic terms), gender, race/ethnicity, income, state of residence, week surveyed, and number of COVID-19-related symptoms experienced in the past 30 days.

^b^ Cohort 2 model adjusted for age, gender, race/ethnicity, level of education, Parkinson’s disease, having or living with someone with comorbidities, and the EQ-5D measure of health status.

Abbreviations: CI, confidence interval; RR, rate ratio.

Based on an examination of descriptive data, three perceived barriers to adherence were included in the C1 model, and one was included in the C2 model (S1 Appendix). In C1 participants, having a child in daycare or school was associated with a 37% increase in contacts (RR 1.37; 95% CI 1.09, 1.72), leaving home for work a 20% increase (RR 1.20; 95% CI 1.01, 1.41), and being concerned about finances a 15% increase (RR 1.15; 95% CI 1.05, 1.25) (Table 2). Leaving home for work had no statistically significant association with contact rate in the fully adjusted model in C2. These findings indicate that among families, perceptions about barriers such as work and childcare were risk factors for greater contact rates.

### Community resilience was associated with increased contacts in the family cohort

Given its importance in response to stressors, we evaluated resilience alongside HBM cognitive factors. Individual resilience was not associated with contact rates in either cohort (Table 2). In the family cohort, each 1-point increase in the 5-point CCRAM community resilience scale was associated with a 26% increase in contacts (RR 1.26; 95% CI 1.11, 1.43). Our results demonstrate that individuals who rated their communities higher on the resilience scale had a greater number of contacts.

### Cognitive factor effects varied based on sociodemographic modifiers

**Fig 2:**
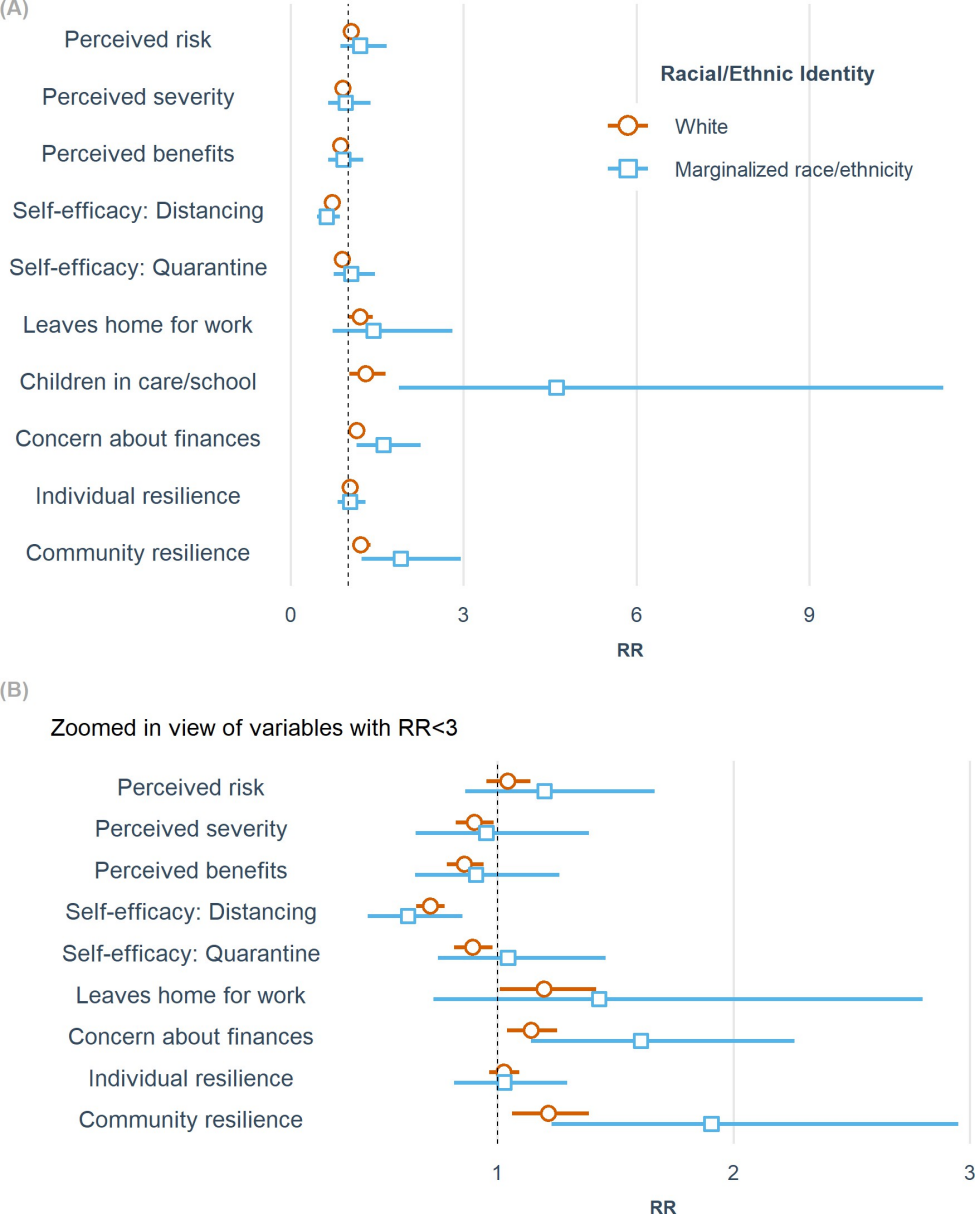
Health Belief Model and resilience constructs stratified by race/ethnicity for the family cohort (C1). (A) shows all constructs. (B) is limited to those variables with rate ratio (RR) estimates <3.0 for closer examination. RRs were estimated from negative binomial models adjusted for age (linear and quadratic terms), gender, income, state of residence, week surveyed, and number of COVID-19-related symptoms experienced in the past 30 days. Bars indicate 95% confidence intervals.

In stratifying our negative binomial models to understand whether the roles of individual perceptions and resilience in physical distancing behavior would differ based on sociodemographic modifiers, we found that the greatest variation was seen in C1 based on race/ethnicity (Table E in S1 Appendix). Among individuals identifying with racially or ethnically marginalized groups, the relative contact rate for those with a child attending daycare or school was 4.61 (95% CI 1.87, 11.32) relative to those whose children were staying home, compared to 1.29 (95% CI 1.02, 1.64) among participants identifying only as white (Fig 2A). Similarly, the effects of community resilience and concern about finances (a perceived barrier) were also magnified in individuals from underrepresented racial/ethnic groups (Fig 2B). Conversely, the protective association of the self-efficacy of quarantine/isolation only existed for white participants. While both marginalized and white participants experienced more contacts associated with community resilience, childcare, and financial concerns, our results show that the effects on individuals identifying with marginalized racial/ethnic groups were substantially larger.

**Fig 3:**
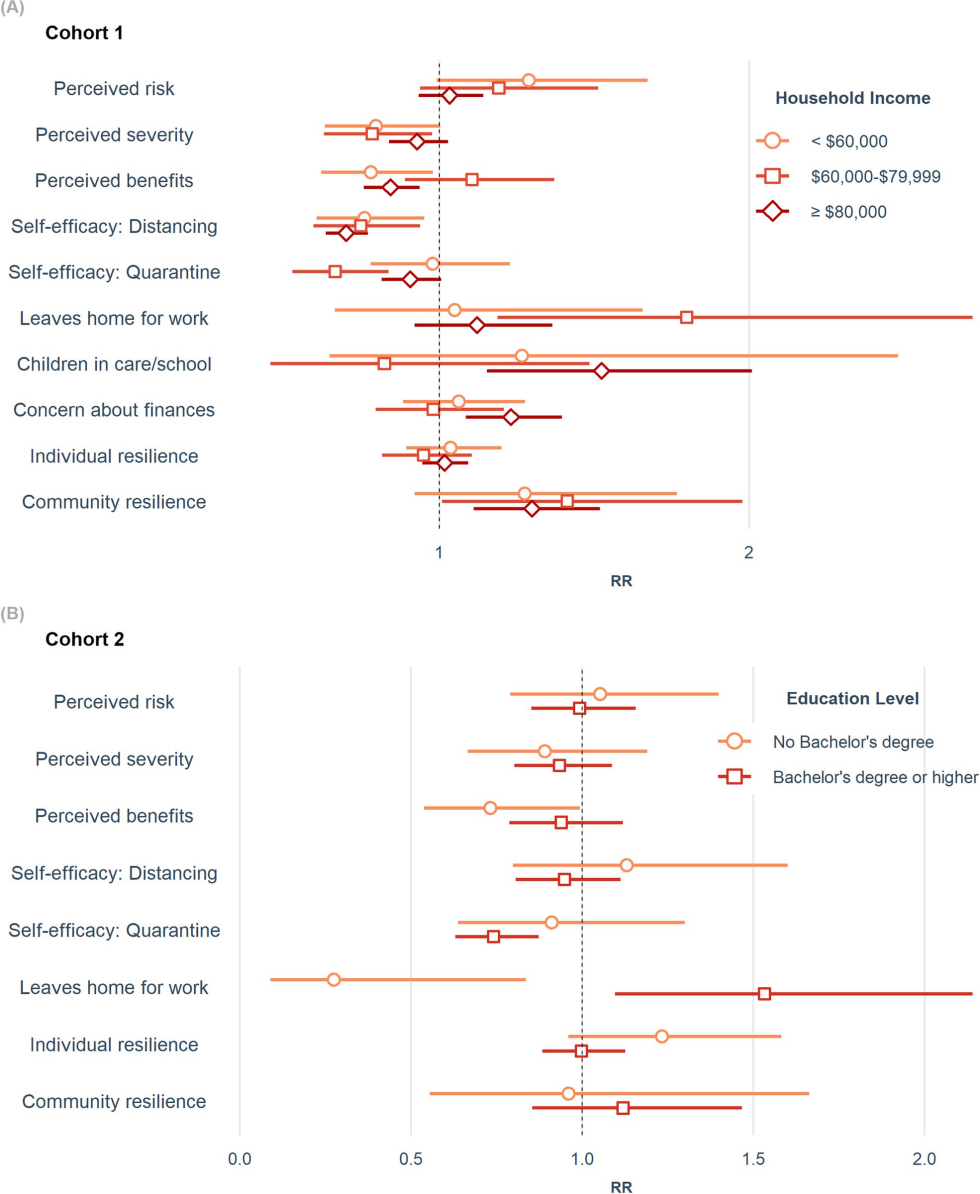
Health Belief Model and resilience constructs stratified by socioeconomic status variables. (A) shows rate ratio (RR) estimates in the family cohort (C1) from negative binomial models by income adjusted for age (linear and quadratic terms), gender, race/ethnicity, state of residence, week surveyed, and number of COVID-19-related symptoms experienced in the past 30 days. (B) shows RR estimates in the older adult cohort (C2) from negative binomial models by education level adjusted for age, gender, race/ethnicity, Parkinson’s disease, having or living with someone with comorbidities, and the EQ-5D measure of health status. Bars indicate 95% confidence intervals.

We examined whether socioeconomic status (SES) modified the effect of perceptions and resilience on contact rates by stratifying our negative binomial models by income in C1 and education in C2. Perceived severity, benefits, the self-efficacy of quarantine/isolation, and all three barriers examined varied by income in C1 (Fig 3A; Table F in S1 Appendix). Particularly, the barriers concern about finances (RR 1.23; 95% CI 1.09, 1.40) and childcare (RR 1.52; 95% CI 1.15, 2.01) were only associated with more contacts among participants with a household income ≥$80,000. The group earning $60,000-$79,999 had the only significant association with leaving home for work (RR 1.80; 95% CI 1.19, 2.72) and the largest association with self-efficacy for quarantine/isolation (RR 0.66; 95% CI 0.53, 0.84).

In C2, the effect of leaving the home for work exhibited qualitative effect modification by level of education (Fig 3B; Table G in S1 Appendix). Participants with a Bachelor’s degree or higher who left the house for work had a 53% *greater* rate of contacts than those who stayed home (RR 1.53; 95% CI 1.10, 2.14), compared to a 73% *lower* contact rate among those who left the house for work (RR 0.27; 95% CI 0.09, 0.84) among those without a Bachelor’s degree. Additionally, the association of self-efficacy for quarantine/isolation was not present for individuals without a Bachelor’s degree, whereas it was only among this group that there was an association with perceived benefits. In both cohorts, these results indicate that the effects of key HBM cognitive factors, particularly perceived barriers to physical distancing adherence, differ by SES.

## Discussion

We sought to identify cognitive factors from the Health Belief Model (HBM) and resilience constructs that could be used to tailor public health messaging for improving adherence to physical distancing in two cohorts that may experience particular but distinct challenges in adhering to physical distancing recommendations. We found that concepts related to self-efficacy were strongly associated with reduced 7-day contact rates in both cohorts. In the family cohort (C1), lower contact rates were also associated with higher perceived severity of COVID-19 and greater perceived benefits of physical distancing, and higher contact rates were associated with greater community resilience and the three barriers we examined.

The construct of self-efficacy has consistently been identified as a strong predictor of many health behaviors [48], including compliance with COVID-19 protective behaviors [38, 42–44, 49]. The two specific aspects of self-efficacy that we measured differed in their impact on adherence to physical distancing based on population. Confidence in the ability to remain 6 feet from others while in public was associated with lower contacts only in the family cohort and persisted across all race/ethnicity and income subgroups examined, which is consistent with another study examining racial/ethnic variation in self-efficacy and COVID-19 protective behaviors [42]. Self-efficacy to quarantine or isolate as necessary was associated with reduced contacts in both cohorts, but these effects were limited to individuals in the higher SES groups. The difference between these two self-efficacy items can be seen crudely as confidence in the ability to stay home when directed (reduce outings) versus, when one does go out, the ability to reduce contacts. Interpreted in this light, our findings suggest that among adults with children, promoting self-efficacy in reducing close contacts is potentially effective across diverse racial, ethnic, and SES groups, even when a person cannot reduce their frequency of outings. Promoting self-efficacy to directly reduce contacts would not likely be effective in older adults. However, promoting self-efficacy to reduce outings could have some benefit in this population, though only in that subset of highly educated older individuals, who are most likely to have the resources to enact recommendations to stay at home.

We used a broad definition of perceptions about barriers to adherence, such as work outside the home and need for childcare, and found that these were of prime importance in the family cohort but not the older adult cohort. Though meta-analyses of HBM constructs have found perceived barriers to be the most consistently powerful of the model’s constructs [48], the COVID-19 literature on protective behaviors reflects our mixed findings [37, 43, 44]. Having a child in daycare or school and leaving the home for work could expose a person to more interactions during transit to childcare/work locations, make them more likely to complete errands once already out, or make the perceived risk of additional contacts beyond those from childcare/work to be negligible by comparison. Individuals concerned about finances could be providing informal childcare for extra income or making multiple, smaller shopping trips to avoid spending large amounts of money at one time. Although these barriers are likely interrelated, the effect estimates we present are adjusted for one another, indicating that all three factors are independently important. All measures were also adjusted for household income and demographic factors, which are frequently associated with physical distancing adherence [19, 24–28], indicating that these factors are likely not responsible for the observed effects of perceived barriers on contact rates. To overcome these barriers to adherence, more work is needed to understand the dominant mechanisms through which they lead to greater contacts, whether they be related to risk perception or the necessary interactions that are part of being out in the world.

Due to the stressful, and potentially even traumatic [50, 51], nature of the pandemic, we felt it necessary to evaluate the role of resilience alongside the HBM. Individual resilience was not associated with contacts in either cohort, overall or within any subpopulation examined. This may be because of the close link between personal resilience and self-efficacy [52], which had a large effect in this study. A primary theoretical distinction between the two constructs is that resiliency can only manifest in the face of a stressor, whereas self-efficacy beliefs are present with or without the stressor [52]. However, the specific aspects of self-efficacy we assessed cannot influence behavior change outside of the stressful circumstances surrounding the pandemic, which may have diluted effects from the self-assessment of general resiliency. We also evaluated the effect of community resilience, which given its buffering role in the wake of natural disasters [36] could be a key resource in enduring a pandemic [53]. Our findings suggest that greater community resilience was associated with increased contacts and that this effect was magnified among individuals identifying with racially or ethnically marginalized groups. Higher perception of community resilience may be associated with stricter mandates, thus helping people feel more comfortable in public. Alternatively, there may be more interactions that stronger, more resilient communities consider essential (e.g. volunteering at a food shelf). Although high community resilience does not appear to contribute to physical distancing adherence, it likely contributes to weathering the pandemic in other ways (e.g. mental health), as resilient communities have many benefits [54, 55].

We evaluated the effects of HBM cognitive factors on physical distancing adherence by race/ethnicity and SES subgroups to better understand what types of message tailoring and other interventions might be most effective in different populations [56]. Our sample size and composition precluded us from examining effects by specific racial and ethnic groups, preventing more nuanced conclusions. As such, our findings should be interpreted as reflecting distinctions due to the social and political consequences [57] of belonging to a marginalized racial or ethnic group in America, generally, not the experience of any particular group. Models stratified by race/ethnicity were adjusted for SES and vice versa to account for the interplay between these factors.

Barriers to adherence, which may be more likely to be socially determined than other perceptions, showed substantial variation by race/ethnicity and SES. Our findings in the family cohort suggest that interventions addressing barriers related to childcare and financial concerns would be more effective in reducing contacts in marginalized than white populations. However, when we stratified by income, only the contact rates of individuals with household incomes ≥$80,000 were adversely affected by these barriers with adequate precision to rule out a null effect. Why concerns about meeting basic financial needs, particularly, would affect only the contact rates of those reporting the highest household incomes is unclear and calls for a more detailed examination of this group’s interactions. In the older adult cohort, leaving home for work appeared to cause individuals without a Bachelor’s degree to be more cautious about their non-work, non-household contacts, whereas those with a Bachelor’s degree appeared to have become less cautious. Education level could be correlated with total wealth accrual and resource availability, which we did not measure directly, and could be motivating more cautious behaviors in those without a college degree.

By examining the number of contacts, we evaluated physical distancing adherence along a quantifiable spectrum of risk, enabling associations to be interpreted as specific reductions in contact rates. During periods of strict physical distancing recommendations, populations are asked to reduce their non-essential outings as much as possible. We did not attempt to differentiate between those (non-work) outings considered essential and those considered non-essential; e.g., three grocery outings in a week may be superfluous for one person but financially necessary for another. We excluded outings for work from our study, because many individuals have less ability to modify whether they go into work than whether they leave the house for other reasons. Rather than expecting complete adherence with physical distancing and abstinence from any unnecessary community contact, our findings provide guidance on how to tailor public health messaging to have the greatest impact on adherence. Tailored messaging, particularly if it can be tailored to multiple theoretical concepts (e.g. cognitive factors), specific behaviors, and demographics, is more effective at changing behavior than untailored messaging or messaging tailored to just one of the above [56]. Among theoretical concepts commonly used in tailored behavior change interventions, self-efficacy, which had large, consistent effects in our study, has been seen to have the greatest effect on behavior change [56].

We selected the HBM as our conceptual model because of its wide historical application in infectious disease behavioral research and recent applications in COVID-19. It has been used to address individual perceptions in order to change behavior, often through risk communication and other public health messaging [48]. This application guided our analysis, in that we chose to analyze the individual constructs of the HBM in a single model to identify those that would be most fruitful to address, controlling for all others. The items we used to measure HBM constructs were specific to COVID-19 and physical distancing, thus customized to the health behavior we were targeting [48].

This analysis was subject to some limitations. As a single cross-sectional survey, we cannot establish the temporal sequence of the factors considered, which may particularly have affected perceived risk and self-efficacy. The family cohort constituted a social media convenience sample, which affected its demographic makeup and could have introduced selection bias. In addition, the study population underrepresented specific racial and ethnic groups, particularly in C2. We have stratified our results by race/ethnicity in C1 to elucidate how our findings may differ by these important factors. While consistent with Minnesota and Iowa median family incomes, a large portion of C1 participants had household incomes ≥$80,000. As income and area-level deprivation have been associated with physical distancing [27, 49], the results of our study may not be generalizable to broader income groups. Social desirability bias in the form of underreporting of outings and contacts was possible, though the survey design attempted to limit this by breaking up the outing/contact types. Similarly, participants may not have recalled all outings/contacts in the past week.

In conclusion, our findings suggest that public health messaging tailored to cognitive factors related to contact rates has the potential to improve pandemic physical distancing adherence, but population-specific needs must be considered to maximize effectiveness. HBM concepts have been used in public health messaging and risk communication to target behavior change; however, this study adds to the growing body of evidence that the impact of messages targeting HBM constructs may not be universal across important modifying factors. Physical distancing adherence will remain an important area to address as long as new SARS-CoV-2 variants continue to evolve and new diseases emerge.

## Supporting information

S1 Appendix Additional methodologic information and supplemental tables. (DOCX)
