# Supplementary material for: Cognitive factors influenced physical distancing adherence during the COVID-19 pandemic in a population-specific way
Source: PLoS One. 2022 May 3;17(5):e0267261. doi: 10.1371/journal.pone.0267261 (PMC9064111; doi:10.1371/journal.pone.0267261)
Supplement: S1 Appendix — (DOCX) [file pone.0267261.s001.docx]

# Supplemental Text

## Outings and Contacts

The seven outing types included: retail, dining, medical facility, caregiving facility, private residence, outdoor, and other outings. Participants indicated their number of outings for the previous week for each type as 0, 1, 2, 3, or more than 3. Retail outings explicitly excluded curbside pick-up that occurred without entering the store. Dining explicitly referred to staying at the establishment to eat or drink.

Contacts were defined as “interactions” to improve recall, and participants were provided with the following examples, “having a conversation, making a purchase, or standing closer than 6 feet to another person for a few minutes.” Outdoor contacts explicitly excluded passing someone on the street without stopping to talk. Participants indicated the number of contacts for each outing type, as well as visitors, as 0, 1, 2, 3, 4, or more than 4.

Outing selections of ‘more than 3’ and contact selections of ‘more than 4’ were treated as 4 and 5, respectively; this assumption was evaluated using a censored Poisson model. We used censored Poisson models adjusted for age, gender, and state [Cohort 1 (C1)] or Parkinson’s disease status [Cohort 2 (C2)] to estimate the true value of ‘greater than 3’ outings and ‘greater than 4’ contacts. The ‘greater than’ values were set to censored, and all other values were treated as uncensored. Based on the model coefficients and combination of covariates, we then calculated the likely value based on a Poisson distribution. After rounding to the nearest integer to obtain a count variable, we compared these estimates to the assumption that ‘greater than 3’ was equal to 4 and ‘greater than 4’ was equal to 5. This assumption was not violated for any of the participants selecting these values, based on the estimates from the censored Poisson; therefore, we proceeded with the simple substitution of 4 outings and 5 contacts in all subsequent analyses.

## Measurement Scales

The Connor-Davidson Resilience Scale (CD-RISC2), which was used to measure individual resilience as part of perceived self-efficacy is a two-item instrument, with each item measured on a scale of 0 to 4 and summed for the overall CD-RISC2 score. In our data, Cronbach’s α = 0.73 for the two items of the CD-RISC2. The ten items of the Conjoint Community Resiliency Assessment Measure (CCRAM), which we used to measure community resilience as part of perceived self-efficacy, were averaged for an overall score between 1 and 5. The CCRAM had a Cronbach’s α = 0.90 in our data.

## Contextual Factors

Participants were surveyed on a range of sociodemographic factors, mental and physical health characteristics, and neighborhood characteristics. Age was measured continuously. Gender was assessed as woman, man, or one of three non-binary categories, which had to be condensed for analysis because of small sample size in them. Participants could indicate any combination of eight race/ethnicity categories, including an ‘other’ category. Household income ranges were provided for participants to select from, ranging from <$20,000 to ≥$80,000 in $20,000 increments. Education levels available for selection included: less than high school; high school or GED; post high school, including trade school; 4 year college degree; and masters/doctorate.

Several variables regarding employment status, type and ownership of the home, household composition, and access to transportation were also assessed. Health-related variables included a visual analog scale rating health from 0 to 100, the EQ-5D 5-item health rating scale, the Kessler-6 scale for assessing stress (1), and several variables specific to COVID-19, including specific symptoms consistent with COVID-19, whether the participant believed they had previously had COVID-19, whether the participant had been tested for COVID-19, and whether any household members suffered from a comorbidity that would increase their likelihood of severe COVID-19 outcomes. Home address was obtained and geocoded to census tract, which was used to assign values from the CDC/ATSDR Social Vulnerability Index (SVI) (2-4) and an index of rurality based on census tract of residence and rural/urban status from the 2010 U.S. Census (5). The week that respondents completed the survey was considered a contextual variable.

## Descriptive Analysis

Perceived barriers to adherence and potential confounding variables were examined in descriptive analyses to determine inclusion in the models for each cohort.

Four possible barriers were assessed, two modeled as continuous variables (Table A) and two dichotomous variables (Tables C and D). The natural spline smoother for financial concerns suggested a relationship with contacts in C1 but not C2. The natural spline smoother for concern about a loved one did not suggest a relationship (i.e. it produced a flat line) with contacts in either cohort. The medians and quartiles differed for having a child in childcare in C1 and leaving home for work in both cohorts. The childcare barrier was not assessed in C2 because there were few participants living with children under the age of 18.

Using the same types of descriptive analyses to evaluate potential confounding variables, we identified the continuous contextual variables survey week, health rating, symptom count, and census tract SVI for inclusion in the C1 model. We also identified a non-linear association between age and contacts. Based on differences in median and/or quartiles, we also included the categorical variables state, public transportation use, and having previously been tested for COVID-19 (Table C). We also determined that income should be used as the socioeconomic status (SES) variable in this cohort. Addition of health rating, SVI, public transportation, and COVID-19 testing to the C1 model either caused >10% missing data or caused the model not to converge; these variables were ultimately excluded from the model.

For the C2 model, we identified the continuous contextual variables EQ-5D health indicator and symptom count for inclusion. We identified the categorical variables having a comorbidity or household member with a comorbidity and believing they had previously had COVID-19 (Table D). The SES variable determined most important for this cohort was education. Addition of symptom count and prior COVID-19 belief either caused >10% missing data or caused the model not to converge; these variables were ultimately excluded from the model.

## Secondary Analysis: Effect Modification

To enable analysis in C1, individuals identifying as American Indian or Alaska Native, Asian, Black, Hispanic/Latinx, Middle Eastern, and Pacific Islander were analyzed together. For the household income strata analyzed in C1, the highest category included those approximately at or above the median household income levels *for families* in Minnesota and Iowa, the middle category included those around the states’ median income levels for *all households*, and the lowest category included those below the median household incomes of both states. Education strata needed to be condensed in C2 for this analysis, to participants with and without a Bachelor’s degree.

# Supplemental Tables

**Table A**. Psychosocial questions and constructs developed for this study.

| Question | Response | Response Value |
| --- | --- | --- |
| Perceived risk:  What do you think is your risk of getting COVID-19?  [scale reversed for analysis] | Very high  High  Moderate  Low  Very low | 1  2  3  4  5 |
| Perceived severity:  If you were to get COVID-19, what do you think is your risk of severe disease or complications?  [scale reversed for analysis] | Very high  High  Moderate  Low  Very low | 1  2  3  4  5 |
| Perceived barrier:  I don’t have enough money to pay for my basic needs right now. | Strongly Disagree  Disagree  Unsure  Agree  Strongly Agree | 1  2  3  4  5 |
| Perceived barrier:  I am concerned about the health of a loved one. | Strongly Disagree  Disagree  Unsure  Agree  Strongly Agree | 1  2  3  4  5 |
| Self-efficacy: During COVID-19 community containment, I am confident that my household has been/is able to: |  |  |
| Stay 6 feet away from non-household members when in public. | Not confident at all  Somewhat confident  Confident  Very confident | 1  2  3  4 |
| Quarantine or isolate yourself (i.e. not leave the house) due to exposure or infection for 14 days, or as directed by public health officials. | Not confident at all  Somewhat confident  Confident  Very confident | 1  2  3  4 |
| Perceived benefits/response efficacy factor (α = 0.91) |  |  |
| Physical/social distancing will protect me from getting COVID-19. | Strongly Disagree  Disagree  Unsure  Agree  Strongly Agree | 1  2  3  4  5 |
| Physical/social distancing will protect my household from getting COVID-19. | Strongly Disagree  Disagree  Unsure  Agree  Strongly Agree | 1  2  3  4  5 |
| Physical/social distancing will protect my community from being overwhelmed by COVID-19. | Strongly Disagree  Disagree  Unsure  Agree  Strongly Agree | 1  2  3  4  5 |

**Table B.** Negative binomial regression output of un-stratified models, including coefficients (not exponentiated), confidence intervals (CIs), standard errors (SEs), and p-values for all variables.

|  | Estimate | 95% CI | SE | P-Value |
| --- | --- | --- | --- | --- |
| ***Cohort 1*** | | | | |
| *Model Covariates* |  | |  |  |
| Risk perception | 0.0646 | -0.0219, 0.1511 | 0.0441 | 0.143 |
| Severity perception | -0.1132 | -0.1991, -0.0273 | 0.0438 | 0.0098 |
| Perceived benefits | -0.1565 | -0.2437, -0.0694 | 0.0444 | <0.001 |
| Self-efficacy: Distancing 6 feet | -0.3450 | -0.4275, -0.2625 | 0.0421 | <0.001 |
| Self-efficacy: Quarantine/isolation | -0.1094 | -0.1972, -0.0217 | 0.0448 | 0.0145 |
| Leaves for work | 0.1799 | 0.0147, 0.3451 | 0.0843 | 0.0328 |
| Children in care/school | 0.3129 | 0.0821, 0.5438 | 0.1178 | 0.0079 |
| Concern about finances | 0.1354 | 0.0452, 0.2256 | 0.046 | 0.0033 |
| Individual resilience | 0.0232 | -0.0360, 0.0824 | 0.0302 | 0.4419 |
| Community resilience | 0.2302 | 0.1029, 0.3574 | 0.0649 | <0.001 |
| Age, per year | 0.1415 | 0.0265, 0.2565 | 0.0587 | 0.0159 |
| Age, per 10 years (squared) | -0.1534 | -0.2903, -0.0164 | 0.0699 | 0.0282 |
| Gender |  |  |  |  |
| Women | 0 | (Ref) |  |  |
| Men | 0.1392 | -0.0887, 0.3670 | 0.1162 | 0.2313 |
| Other gender | 0.1504 | -1.1366, 1.4374 | 0.6566 | 0.8189 |
| Marginalized race/ethnicity | 0.0774 | -0.1956, 0.3505 | 0.1393 | 0.5782 |
| Household income |  |  |  |  |
| <$60,000 | 0.1298 | -0.1023, 0.3619 | 0.1184 | 0.2731 |
| $60,000 - $79,999 | 0.0537 | -0.1634, 0.2708 | 0.1108 | 0.6276 |
| ≥$80,000 | 0 | (Ref) |  |  |
| Week surveyed | 0.0975 | 0.0488, 0.1462 | 0.0248 | <0.001 |
| Lives in Iowa (vs. MN) | 0.1087 | -0.0468, 0.2643 | 0.0794 | 0.1707 |
| Number of COVID-19 symptoms | 0.0580 | -0.0036, 0.1196 | 0.0314 | 0.0651 |
| *Model Statistics* |  |  |  |  |
| N | 925 |  |  |  |
| Pseudo R^2^ | 0.223 |  |  |  |
| ***Cohort 2*** | | | | |
| *Model Covariates* |  | |  |  |
| Risk perception | -0.0343 | -0.1701, 0.1015 | 0.0693 | 0.6208 |
| Severity perception | -0.0661 | -0.2018, 0.0695 | 0.0692 | 0.3394 |
| Perceived benefits | -0.1479 | -0.2997, 0.0038 | 0.0774 | 0.0561 |
| Self-efficacy: Distancing 6 feet | -0.0506 | -0.1966, 0.0954 | 0.0745 | 0.4971 |
| Self-efficacy: Quarantine/isolation | -0.2643 | -0.4174, -0.1112 | 0.0781 | <0.001 |
| Leaves for work | 0.2488 | -0.0831, 0.5807 | 0.1693 | 0.1417 |
| Individual resilience | 0.0342 | -0.0713, 0.1397 | 0.0538 | 0.5248 |
| Community resilience | 0.0860 | -0.1540, 0.3259 | 0.1224 | 0.4826 |
| Age, per year | -0.0178 | -0.0344, -0.0011 | 0.0085 | 0.0362 |
| Men | 0.0539 | -0.1753, 0.2832 | 0.117 | 0.6447 |
| Marginalized race/ethnicity | -1.5766 | -2.8437, -0.3096 | 0.6465 | 0.0147 |
| Parkinson’s disease | 0.2813 | 0.0094, 0.5532 | 0.1387 | 0.0426 |
| Education level |  |  |  |  |
| High school or less | -0.4760 | -1.1598, 0.2078 | 0.3489 | 0.1724 |
| Some college or 2-year degree | 0.2735 | 0.0121, 0.5349 | 0.1334 | 0.0403 |
| Bachelor’s degree or more | 0 | (Ref) |  |  |
| Person with comorbidity in household | 0.4204 | 0.1748, 0.6660 | 0.1253 | <0.001 |
| EQ-5D health status index | 0.8821 | -0.1350, 1.8993 | 0.519 | 0.0892 |
| *Model Statistics* |  |  |  |  |
| N | 309 |  |  |  |
| Pseudo R^2^ | 0.165 |  |  |  |

**Table C**. Family cohort (C1) descriptive analysis of median non-work, non-household contacts by categorical Health Belief Model barriers and adjustment variables.

|  | Median | 25^th^ Quantile | 75^th^ Quantile |
| --- | --- | --- | --- |
| POTENTIAL BARRIERS | | | |
| Leaves home for work |  |  |  |
| No | 4 | 1 | 9 |
| Yes | 6 | 2 | 14 |
| Children in daycare/school |  |  |  |
| No | 4 | 1 | 10 |
| Yes | 7 | 3 | 14 |
| POTENTIAL ADJUSTMENT FACTORS | | | |
| Gender identity |  |  |  |
| Woman | 4 | 1 | 10 |
| Man | 6 | 2 | 12 |
| Transgender or Non-binary | 4 | 2 | 9 |
| Racial/ethnic identity |  |  |  |
| White only | 4 | 1 | 10 |
| Marginalized race/ethnicity | 4 | 1 | 10 |
| Employment status |  |  |  |
| Employed full-time | 4 | 1 | 11 |
| Employed part-time | 4 | 1 | 9 |
| Not employed | 4 | 1 | 8 |
| Level of education |  |  |  |
| High school or less | 5 | 2 | 12 |
| Some college or 2-year degree | 5 | 2 | 12 |
| Bachelor’s degree or higher | 4 | 1 | 10 |
| Income |  |  |  |
| Under $60,000 | 5.5 | 2 | 12 |
| $60,000-$79,999 | 5 | 1 | 11 |
| $80,000 or above | 4 | 1 | 10 |
| Home ownership |  |  |  |
| Own | 4 | 1 | 10 |
| Rent | 5 | 2 | 10.5 |
| Type of home |  |  |  |
| Stand-alone home | 4 | 1 | 10 |
| Duplex, quadruplex, or townhouse | 4.5 | 1 | 11.25 |
| Apartment, condo, or group home | 5 | 1.75 | 10 |
| State |  |  |  |
| Iowa | 5 | 1 | 12 |
| Minnesota | 4 | 1 | 9 |
| Uses public transportation |  |  |  |
| No | 4 | 1 | 10.75 |
| Yes | 2 | 0.25 | 6 |
| Self/household member has comorbidity |  |  |  |
| No | 4 | 1 | 10 |
| Yes | 4 | 1 | 10 |
| Believes previously had COVID-19 |  |  |  |
| No | 4 | 1 | 10 |
| Yes | 5 | 2 | 12 |
| Has been tested for COVID-19 |  |  |  |
| No | 4 | 1 | 10 |
| Yes | 7 | 1 | 13.25 |

**Table D**. Older adult cohort (C2) descriptive analysis of median non-work, non-household contacts by categorical Health Belief Model barriers and adjustment variables.

|  | Median | 25^th^ Quantile | 75^th^ Quantile |
| --- | --- | --- | --- |
| POTENTIAL BARRIERS | | | |
| Leaves home for work |  |  |  |
| No | 4 | 2 | 9 |
| Yes | 6 | 2 | 12.5 |
| POTENTIAL ADJUSTMENT FACTORS | | | |
| Gender identity |  |  |  |
| Woman | 4 | 2 | 9 |
| Man | 5 | 2 | 10 |
| Transgender or Non-binary | 0 | 0 | 0 |
| Racial/ethnic identity |  |  |  |
| White only | 4 | 2 | 9.75 |
| Marginalized race/ethnicity | 1 | 0.25 | 1.75 |
| Employment status |  |  |  |
| Employed full-time | 5 | 1 | 9 |
| Employed part-time | 7.5 | 2 | 10 |
| Not employed | 4 | 2 | 9 |
| Level of education |  |  |  |
| High school or less | 2 | 2 | 5 |
| Some college or 2-year degree | 4 | 1 | 11.25 |
| Bachelor’s degree or higher | 4.5 | 2 | 9 |
| Income |  |  |  |
| Under $60,000 | 3 | 2 | 8 |
| $60,000-$79,999 | 5 | 2 | 12.5 |
| $80,000 or above | 4 | 2 | 10 |
| Home ownership |  |  |  |
| Own | 4 | 2 | 9 |
| Rent | 5 | 3.75 | 10.75 |
| Type of home |  |  |  |
| Stand-alone home | 4 | 2 | 10 |
| Duplex, quadruplex, or townhouse | 6 | 2.25 | 11.75 |
| Apartment, condo, or group home | 4 | 2 | 7 |
| Parkinson’s disease |  |  |  |
| No | 4 | 2 | 8 |
| Yes | 5 | 2 | 11 |
| Lives alone |  |  |  |
| No | 4 | 2 | 9.75 |
| Yes | 4 | 2 | 8 |
| Access to own transportation |  |  |  |
| No | 3 | 2 | 5 |
| Yes | 4 | 2 | 9 |
| Uses public transportation |  |  |  |
| No | 4 | 2 | 9 |
| Yes | 4 | 2 | 10 |
| Self/household member has comorbidity |  |  |  |
| No | 4 | 2 | 8.5 |
| Yes | 4.5 | 2 | 10 |
| Believes previously had COVID-19 |  |  |  |
| No | 4 | 2 | 9.5 |
| Yes | 1.5 | 0.75 | 2.25 |

**Table E.** Negative binomial regression output of Cohort 1 models stratified by race/ethnicity, including coefficients (not exponentiated), confidence intervals (CIs), standard errors (SEs), and p-values for all variables.

|  | Estimate | 95% CI | SE | P-Value | Estimate | 95% CI | SE | P-Value |
| --- | --- | --- | --- | --- | --- | --- | --- | --- |
|  | ***Identifies with Marginalized Race/Ethnicity*** | | | | ***Identifies Only as White*** | | | |
| *Model Covariates* |  |  |  |  |  |  |  |  |
| Risk perception | 0.181 | (-0.148, 0.511) | 0.168 | 0.280 | 0.041 | (-0.049, 0.131) | 0.046 | 0.371 |
| Severity perception | -0.050 | (-0.427, 0.328) | 0.193 | 0.797 | -0.105 | (-0.194, -0.016) | 0.045 | 0.020 |
| Perceived benefits | -0.098 | (-0.428, 0.233) | 0.169 | 0.563 | -0.152 | (-0.243, -0.061) | 0.046 | 0.001 |
| Self-efficacy: Distancing 6 feet | -0.478 | (-0.796, -0.161) | 0.162 | 0.003 | -0.338 | (-0.423, -0.253) | 0.044 | <0.001 |
| Self-efficacy: Quarantine/isolation | 0.043 | (-0.292, 0.377) | 0.171 | 0.803 | -0.113 | (-0.204, -0.022) | 0.046 | 0.015 |
| Leaves for work | 0.357 | (-0.316, 1.030) | 0.343 | 0.299 | 0.179 | (0.009, 0.350) | 0.087 | 0.039 |
| Children in care/school | 1.528 | (0.628, 2.427) | 0.459 | 0.001 | 0.258 | (0.019, 0.497) | 0.122 | 0.034 |
| Concern about finances | 0.474 | (0.133, 0.814) | 0.174 | 0.006 | 0.132 | (0.038, 0.225) | 0.048 | 0.006 |
| Individual resilience | 0.027 | (-0.205, 0.258) | 0.118 | 0.820 | 0.026 | (-0.035, 0.088) | 0.031 | 0.403 |
| Community resilience | 0.644 | (0.207, 1.082) | 0.223 | 0.004 | 0.194 | (0.060, 0.327) | 0.068 | 0.005 |
| Age, per year | -0.032 | (-0.539, 0.474) | 0.258 | 0.900 | 0.162 | (0.043, 0.282) | 0.061 | 0.008 |
| Age, per 10 years (squared) | 0.117 | (-0.501, 0.735) | 0.315 | 0.710 | -0.182 | (-0.324, -0.040) | 0.072 | 0.012 |
| Gender |  |  |  |  |  |  |  |  |
| Women | 0 | (Ref) |  |  | 0 | (Ref) |  |  |
| Men | 0.656 | (-0.089, 1.401) | 0.380 | 0.084 | 0.083 | (-0.156, 0.322) | 0.122 | 0.496 |
| Other gender | N/A |  |  |  | 0.166 | (-1.116, 1.448) | 0.654 | 0.800 |
| Household income |  |  |  |  |  |  |  |  |
| <$60,000 | -0.333 | (-1.265, 0.599) | 0.476 | 0.484 | 0.160 | (-0.080, 0.401) | 0.123 | 0.191 |
| $60,000 - $79,999 | 0.087 | (-0.723, 0.896) | 0.413 | 0.834 | 0.032 | (-0.195, 0.258) | 0.115 | 0.785 |
| ≥$80,000 | 0 | (Ref) |  |  | 0 | (Ref) |  |  |
| Week surveyed | 0.206 | (0.032, 0.379) | 0.089 | 0.020 | 0.097 | (0.047, 0.148) | 0.026 | <0.001 |
| Lives in Iowa (vs. MN) | -0.424 | (-1.068, 0.219) | 0.328 | 0.196 | 0.136 | (-0.025, 0.297) | 0.082 | 0.097 |
| Number of COVID-19 symptoms | 0.067 | (-0.225, 0.360) | 0.149 | 0.652 | 0.047 | (-0.017, 0.110) | 0.032 | 0.150 |
| *Model Statistics* |  |  |  |  |  |  |  |  |
| N | 70 |  |  |  | 855 |  |  |  |
| Pseudo R^2^ | 0.476 |  |  |  | 0.217 |  |  |  |

**Table F.** Negative binomial regression output of Cohort 1 models stratified by income, including coefficient estimates (not exponentiated), confidence intervals (CIs), standard errors (SEs), and p-values for all variables.

|  | Est. | 95% CI | SE | P-Val | Est. | 95% CI | SE | P-VaL | Est. | 95% CI | SE | P-Val |
| --- | --- | --- | --- | --- | --- | --- | --- | --- | --- | --- | --- | --- |
|  | ***< $60,000*** | | | | ***$60,000-$79,999*** | | | | ***≥$80,000*** | | | |
| *Model Covariates* |  |  |  |  |  |  |  |  |  |  |  |  |
| Risk perception | 0.253 | (-0.008, 0.514) | 0.133 | 0.058 | 0.175 | (-0.064, 0.414) | 0.122 | 0.151 | 0.032 | (-0.069, 0.133) | 0.051 | 0.534 |
| Severity perception | -0.229 | (-0.461, 0.003) | 0.118 | 0.053 | -0.245 | (-0.466, -0.024) | 0.113 | 0.030 | -0.074 | (-0.177, 0.028) | 0.052 | 0.157 |
| Perceived benefits | -0.250 | (-0.480, -0.020) | 0.117 | 0.033 | 0.099 | (-0.116, 0.315) | 0.110 | 0.367 | -0.173 | (-0.279, -0.066) | 0.054 | 0.002 |
| Self-efficacy: Distancing 6 feet | -0.277 | (-0.504, -0.050) | 0.116 | 0.017 | -0.292 | (-0.520, -0.064) | 0.116 | 0.012 | -0.358 | (-0.455, -0.262) | 0.049 | <0.001 |
| Self-efficacy: Quarantine/isolation | -0.022 | (-0.250, 0.205) | 0.116 | 0.849 | -0.411 | (-0.643, -0.179) | 0.118 | 0.001 | -0.100 | (-0.205, 0.006) | 0.054 | 0.064 |
| Leaves for work | 0.047 | (-0.410, 0.504) | 0.233 | 0.839 | 0.587 | (0.172, 1.001) | 0.211 | 0.006 | 0.114 | (-0.083, 0.311) | 0.100 | 0.256 |
| Children in care/school | 0.235 | (-0.438, 0.908) | 0.343 | 0.493 | -0.196 | (-0.787, 0.396) | 0.302 | 0.516 | 0.420 | (0.143, 0.698) | 0.141 | 0.003 |
| Concern about finances | 0.060 | (-0.124, 0.244) | 0.094 | 0.522 | -0.020 | (-0.229, 0.189) | 0.107 | 0.851 | 0.208 | (0.083, 0.333) | 0.064 | 0.001 |
| Individual resilience | 0.036 | (-0.112, 0.183) | 0.075 | 0.636 | -0.052 | (-0.204, 0.100) | 0.078 | 0.499 | 0.016 | (-0.057, 0.089) | 0.037 | 0.667 |
| Community resilience | 0.243 | (-0.083, 0.569) | 0.166 | 0.144 | 0.345 | (0.009, 0.682) | 0.172 | 0.044 | 0.261 | (0.105, 0.417) | 0.080 | 0.001 |
| Age, per year | 0.199 | (-0.044, 0.443) | 0.124 | 0.109 | 0.183 | (-0.073, 0.440) | 0.131 | 0.162 | 0.133 | (-0.020, 0.286) | 0.078 | 0.089 |
| Age, per 10 years (squared) | -0.212 | (-0.505, 0.080) | 0.149 | 0.155 | -0.206 | (-0.518, 0.107) | 0.159 | 0.197 | -0.143 | (-0.323, 0.038) | 0.092 | 0.121 |
| Gender |  |  |  |  |  |  |  |  |  |  |  |  |
| Women | 0 | (Ref) |  |  | 0 | (Ref) |  |  | 0 | (Ref) |  |  |
| Men | -0.003 | (-0.915, 0.908) | 0.465 | 0.994 | 0.235 | (-0.371, 0.842) | 0.309 | 0.447 | 0.132 | (-0.123, 0.387) | 0.130 | 0.310 |
| Other gender | N/A |  |  |  | -0.007 | (-2.318, 2.304) | 1.179 | 0.995 | 0.299 | (-1.237, 1.834) | 0.783 | 0.703 |
| Marginalized race/ethnicity | -0.009 | (-0.725, 0.707) | 0.365 | 0.981 | 0.266 | (-0.390, 0.922) | 0.335 | 0.427 | 0.091 | (-0.240, 0.422) | 0.169 | 0.589 |
| Week surveyed | 0.055 | (-0.088, 0.199) | 0.073 | 0.452 | -0.009 | (-0.142, 0.124) | 0.068 | 0.895 | 0.126 | (0.070, 0.183) | 0.029 | <0.001 |
| Lives in Iowa (vs. MN) | -0.013 | (-0.415, 0.389) | 0.205 | 0.950 | 0.486 | (0.079, 0.893) | 0.208 | 0.019 | 0.137 | (-0.050, 0.324) | 0.095 | 0.151 |
| Number of COVID-19 symptoms | 0.094 | (-0.046, 0.235) | 0.072 | 0.187 | 0.030 | (-0.117, 0.177) | 0.075 | 0.692 | 0.049 | (-0.030, 0.129) | 0.041 | 0.226 |
| *Model Statistics* |  |  |  |  |  |  |  |  |  |  |  |  |
| N | 125 |  |  |  | 129 |  |  |  | 671 |  |  |  |
| Pseudo R^2^ | 0.240 |  |  |  | 0.342 |  |  |  | 0.227 |  |  |  |

**Table G.** Negative binomial regression output of Cohort 2 models stratified by education level, including coefficients (not exponentiated), confidence intervals (CIs), standard errors (SEs), and p-values for all variables.

|  | Estimate | 95% CI | SE | P-Value | Estimate | 95% CI | SE | P-Value |
| --- | --- | --- | --- | --- | --- | --- | --- | --- |
|  | ***No Bachelor’s Degree*** | | | | ***Bachelor’s Degree*** | | | |
| *Model Covariates* |  |  |  |  |  |  |  |  |
| Risk perception | 0.050 | (-0.235, 0.336) | 0.146 | 0.729 | -0.007 | (-0.160, 0.145) | 0.078 | 0.924 |
| Severity perception | -0.116 | (-0.406, 0.174) | 0.148 | 0.433 | -0.069 | (-0.221, 0.083) | 0.077 | 0.374 |
| Perceived benefits | -0.313 | (-0.619, -0.007) | 0.156 | 0.045 | -0.064 | (-0.240, 0.112) | 0.090 | 0.478 |
| Self-efficacy: Distancing 6 feet | 0.122 | (-0.225, 0.470) | 0.177 | 0.49 | -0.054 | (-0.215, 0.107) | 0.082 | 0.511 |
| Self-efficacy: Quarantine/isolation | -0.094 | (-0.450, 0.262) | 0.182 | 0.605 | -0.300 | (-0.463, -0.136) | 0.084 | <0.001 |
| Leaves for work | -1.298 | (-2.416, -0.179) | 0.571 | 0.023 | 0.427 | (0.092, 0.761) | 0.171 | 0.012 |
| Individual resilience | 0.209 | (-0.040, 0.459) | 0.127 | 0.100 | -0.002 | (-0.123, 0.118) | 0.061 | 0.969 |
| Community resilience | -0.041 | (-0.590, 0.509) | 0.280 | 0.885 | 0.113 | (-0.158, 0.383) | 0.138 | 0.413 |
| Age, per year | -0.024 | (-0.057, 0.009) | 0.017 | 0.158 | -0.015 | (-0.034, 0.004) | 0.010 | 0.115 |
| Gender |  |  |  |  |  |  |  |  |
| Women | 0 | (Ref) |  |  | 0 | (Ref) |  |  |
| Men | 0.007 | (-0.555, 0.569) | 0.287 | 0.980 | 0.066 | (-0.179, 0.310) | 0.125 | 0.598 |
| Marginalized race/ethnicity | -1.398 | (-3.455, 0.659) | 1.049 | 0.183 | -1.269 | (-2.897, 0.360) | 0.831 | 0.127 |
| Parkinson’s disease | 0.574 | (-0.057, 1.206) | 0.322 | 0.074 | 0.230 | (-0.067, 0.528) | 0.152 | 0.130 |
| Person with comorbidity in household | 0.840 | (0.253, 1.426) | 0.299 | 0.005 | 0.313 | (0.044, 0.583) | 0.137 | 0.023 |
| EQ-5D health status index | 1.493 | (-1.107, 4.094) | 1.327 | 0.260 | 0.439 | (-0.653, 1.532) | 0.558 | 0.431 |
| *Model Statistics* |  |  |  |  |  |  |  |  |
| N | 80 |  |  |  | 229 |  |  |  |
| Pseudo R^2^ | 0.304 |  |  |  | 0.154 |  |  |  |

# Supplemental References

1. Kessler RC, Barker PR, Colpe LJ, Epstein JF, Gfroerer JC, Hiripi E, et al. Screening for serious mental illness in the general population. Arch Gen Psychiatry. 2003;60(2):184-9.

2. Flanagan BE, Gregory EW, Hallisey EJ, Heitgerd JL, Lewis B. A Social Vulnerability Index for Disaster Management. Journal of Homeland Security and Emergency Management. 2011;8(1):1-22.

3. Flanagan BE, Hallisey EJ, Adams E, Lavery A. Measuring Community Vulnerability to Natural and Anthropogenic Hazards: The Centers for Disease Control and Prevention's Social Vulnerability Index. J Environ Health. 2018;80(10):34-6.

4. Centers for Disease Control and Prevention, Agency for Toxic Substances and Disease Registry. Social Vulnerability Index. Geospatial Research, Analysis, and Services Program. U.S.2018.

5. Manson S, Schroeder J, Van Riper D, Kugler T, Ruggles S. IPUMS National Historical Geographic Information System. 16.0 ed. Minneapolis, MN: IPUMS; 2019.
